# Value of Multimodality Imaging in the Early Diagnosis of Intravascular and Intracardiac Leiomyomatosis

**DOI:** 10.1016/j.jaccas.2026.106874

**Published:** 2026-03-20

**Authors:** Yufei Xiang, Yu Kang, Rongkai Yan, Devicka Ojha, Samy Ataya, James J. Yun, Venkatesh Krishnamurthi, Stephen Waggoner

**Affiliations:** a Department of Internal Medicine, The Ohio State Wexner Medical Center, Columbus, Ohio, USA; b Division of Cardiovascular Medicine, Department of Internal Medicine, The Ohio State Wexner Medical Center, Columbus, Ohio, USA; c Department of Radiology, The Ohio State Wexner Medical Center, Columbus, Ohio, USA; d Department of Cardiothoracic Surgery, Cleveland Clinic, Cleveland, Ohio, USA; e Glickman Urologic Institute, Cleveland Clinic, Cleveland, Ohio, USA; f Department of Gynecologic Surgical Oncology, Cleveland Clinic, Cleveland, Ohio, USA

**Keywords:** intracardiac tumor, intravascular tumor, multimodality imaging, leiomyomatosis

## Abstract

**Background:**

Intravenous and intracardiac leiomyomatosis (IVL/ICL) are rare benign smooth-muscle tumors that can extend from pelvic veins into the right heart, often mimicking thrombus or malignancy.

**Case Summary:**

A 51-year-old woman with uterine fibroids was referred for evaluation of a right atrial mass initially suspected to be thrombus. Multimodality imaging, including echocardiography, computed tomography, magnetic resonance imaging, positron emission tomography, venography, and intravascular ultrasound, identified a highly vascular intraluminal mass extending from the right gonadal vein through the inferior vena cava into the right atrium, consistent with IVL/ICL. She underwent single-stage en bloc resection, and pathology confirmed a benign leiomyoma.

**Discussion:**

This case highlights the pivotal role of multimodality imaging in early diagnosis and multidisciplinary surgical planning, preventing treatment delay and improving outcomes.

**Take-Home Message:**

Multimodality imaging is essential for early and accurate diagnosis of IVL/ICL and for guiding safe, complete resection.

## Graphical Abstract

**Figure:**
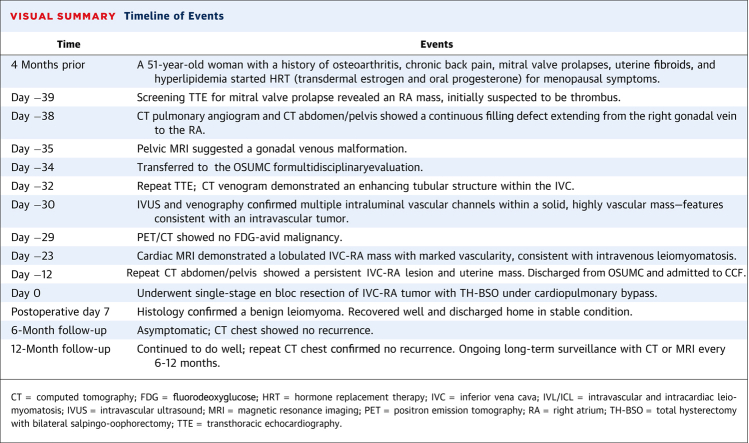

## History of Presentation

A 51-year-old woman with a history of osteoarthritis, chronic back pain from a herniated disc, dysplastic nevi, mitral valve prolapse, uterine fibroids, and hyperlipidemia was transferred from an outside hospital for evaluation of a possible right atrial (RA) and inferior vena cava (IVC) thrombus versus tumor. She had a strong family history of breast cancer and had initiated hormone replacement therapy with transdermal estrogen and oral progesterone approximately 4 months earlier for menopausal symptoms.

The abnormal finding was discovered incidentally during a screening transthoracic echocardiography (TTE) for her known mitral valve prolapse at the outside hospital. Subsequent computed tomography (CT) pulmonary angiography, IVC venography, and follow-up chest and abdominal magnetic resonance imaging (MRI) revealed a filling defect extending from the gonadal vein to the RA, raising concern for thromboembolism versus malignancy. She was started on intravenous heparin therapy.

The patient was largely asymptomatic with a negative review of systems, outside of intermittent palpitations. On admission, vital signs were stable, and physical examination revealed no jugular venous distention, peripheral edema, or cardiac murmurs.

## Past Medical History

The patient's medical history included osteoarthritis, chronic lumbar disc disease, mitral valve prolapse, uterine leiomyoma with hypertrophy of the uterus, fibrocystic breast disease, hyperlipidemia, obesity, and seasonal allergies. She had a remote history of condyloma acuminatum, colon polyps, and dysplastic nevi, all treated or removed. She had no prior thromboembolic, malignant, or autoimmune disease.

Surgical history included bilateral breast lumpectomies for benign cystic mastopathy (2014 and 2019), laparoscopic cholecystectomy (2001), intrauterine device insertion and removal, and mole excision. She was taking transdermal estrogen and oral progesterone for postmenopausal symptoms.

She was a former smoker who quit 17 years earlier, drank alcohol occasionally, and denied illicit drug use. Her family history was notable for hypertension and heart disease in her father, diabetes in both grandparental lineages, and breast and prostate cancer in relatives.

## Differential Diagnosis

Initial considerations included the following:

• Thromboembolism, given the intraluminal IVC defect and recent estrogen therapy.
• Malignant tumor thrombus with possible renal cell or uterine origin.
• RA myxoma, arising from the interatrial septum and not continuous with the venous system.
• Intravascular and intracardiac leiomyomatosis (IVL/ICL), giving continuity with pelvic veins and history of uterine fibroids.
• Lipomatous masses (lipoma or liposarcoma).

## Investigations

**Figure 1:**
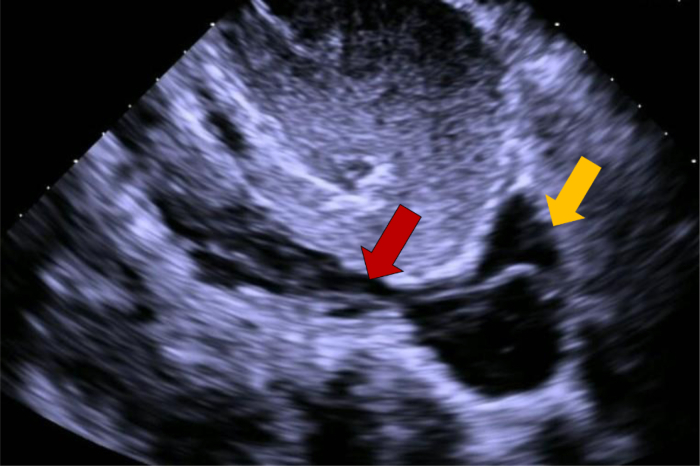
Transthoracic Echocardiography Findings TTE reveals a mobile echogenic mass within the right atrium, which intermittently prolapses toward the tricuspid valve during diastole. The red arrow highlights a tubular structure extending from the IVC into the atrial chamber, consistent with a tumor thrombus or an extension of intravascular leiomyomatosis. The yellow arrow indicates the right atrial cavity. Additional dynamic imaging is provided in Video 1, Video 2, Video 3, Video 4. IVC = inferior vena cava; RA = right atrium; TTE = transthoracic echocardiography.

**Figure 2:**
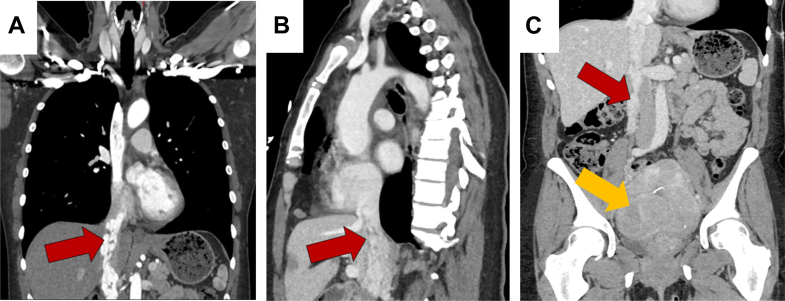
CT Pulmonary Angiogram and CT of the Abdomen/Pelvis Contrast-enhanced CT pulmonary angiogram (A) early phase and (B) late phase, and (C) CT of the abdomen and pelvis demonstrating an extensive intraluminal filling defect extending from the right gonadal vein into the IVC and RA. The red arrow indicates extensive tumor involvement within the IVC, and the yellow arrow denotes the uterine leiomyoma. CT = computed tomography; IVC = inferior vena cava; RA = right atrium.

**Figure 3:**
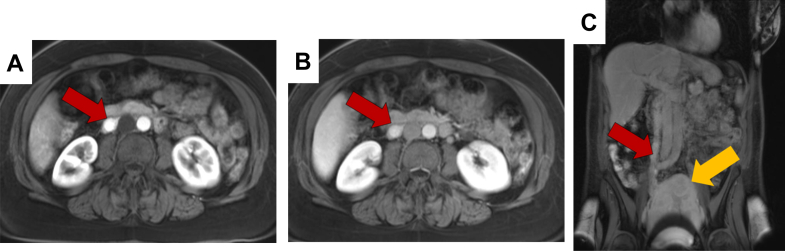
MRI in Arterial, Venous, and Delayed Postcontrast Phases MRI axial (A) arterial and (B) venous postcontrast phase images show an enhancing intraluminal mass (red arrows) within the IVC. (C) Coronal delayed postcontrast phase image demonstrates a heterogeneously enhancing lesion extending along the IVC and contiguous with the right gonadal vein. Persistent enhancement throughout the mass indicates high vascularity, consistent with a fibroid origin. The red arrow marks the intravascular tumor component, and the yellow arrow identifies the uterine leiomyoma. CT = computed tomography; IVC = inferior vena cava; MRI = magnetic resonance imaging; RA = right atrium.

At the outside hospital, transthoracic echocardiography, CT angiography, and pelvic MRI demonstrated a continuous intraluminal filling defect extending from the right gonadal vein into the RA, raising concern for thrombus versus tumor. The patient was transferred to the Ohio State University Wexner Medical Center for multidisciplinary evaluation. Review by our radiology team confirmed a mobile echogenic mass within the RA on echocardiography (Figure 1, Video 1, Video 2, Video 3, Video 4) and a lobulated, enhancing structure on CT (Figure 2) and MRI (Figure 3) tracking contiguously from the right gonadal vein through the IVC into the RA.

**Figure 4:**
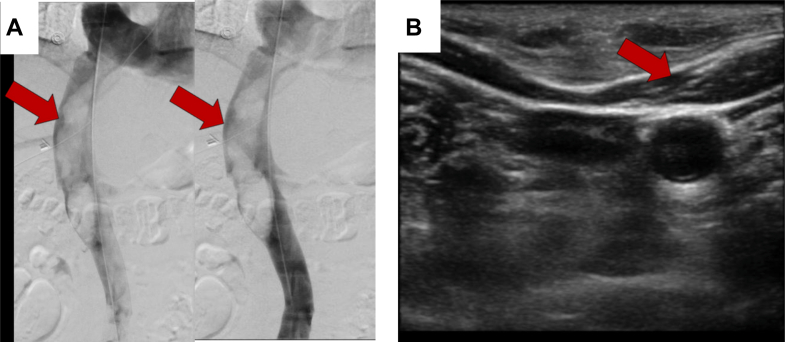
Venography and Intravascular Ultrasound Findings (A) Venography shows a large expansile intraluminal filling defect within the IVC (red arrow), extending from just below the renal veins to the cavoatrial junction. (B) IVUS demonstrates multiple intraluminal vascular channels without endothelial invasion or adherence, indicating a vascular intraluminal tumor rather than thrombus. These findings support the diagnosis of intravenous leiomyomatosis. Additional dynamic imaging is provided in Videos 5 and 6. IVC = inferior vena cava; IVUS = intravascular ultrasound.

**Figure 5:**
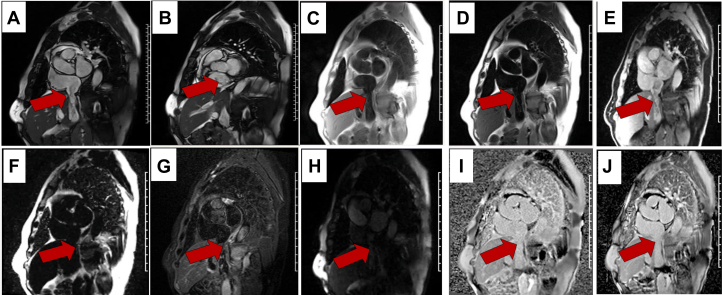
Cardiac MRI Findings Cardiac MRI demonstrating a lobulated, spongy intraluminal mass (red arrows) extending from the IVC into the RA, without evidence of myocardial invasion. (A and B) Cine steady-state free-precession (SSFP) images and (C) T1-weighted and (D) T2-weighted sequences show a mobile, T1- and T2-isointense mass arising from the IVC (red arrow in A) and projecting into the RA (red arrow in B), clearly separate from the atrial wall. Dixon (E) water-only and (F) fat-only images, along with the absence of signal suppression on (G) T2-weighted short tau inversion recovery (STIR), confirmed the lack of macroscopic fat within the lesion. (H) First-pass perfusion imaging demonstrated rapid, avid enhancement, indicating high vascularity and effectively excluding thrombus. Both (I) phase-sensitive inversion recovery (PSIR) LGE imaging and (J) long-TI imaging showed complete gadolinium enhancement of the IVC and RA mass, indicating the absence of fibrosis, necrosis, or myocardial infiltration and supporting tissue characterization consistent with tumor rather than bland thrombus. IVC = inferior vena cava; LGE = late gadolinium enhancement; MRI = magnetic resonance imaging; RA = right atrium.

**Figure 6:**
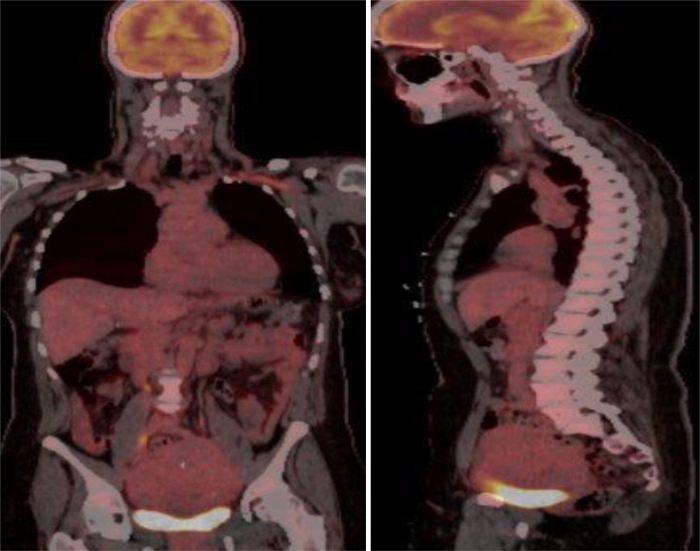
Whole-Body PET/CT Findings PET/CT demonstrating absence of fluorodeoxyglucose (FDG) uptake within the IVC and RA mass, supporting a benign process making a metabolically malignancy less likely. IVC = inferior vena cava; PET/CT = positron emission tomography/computed tomography; RA = right atrium.

Given the uncertain nature of the lesion, systemic anticoagulation was initiated in our hospital, and consultations were obtained from vascular surgery, cardiothoracic surgery, cardiology, gynecologic oncology, and interventional radiology. Comprehensive multimodality imaging was subsequently performed. Venography revealed a large, expansile intraluminal filling defect extending from just below the renal veins to the cavoatrial junction (Figure 4, Video 5). Intravascular ultrasound (IVUS) demonstrated multiple intraluminal vascular channels without endothelial invasion—features consistent with a vascular intraluminal tumor rather than thrombus (Figure 4, Video 6). Cardiac MRI (Figure 5) showed a lobulated, spongy, T1- and T2-isointense mass extending from the IVC into the RA, distinct from the atrial wall and without myocardial invasion. First-pass perfusion revealed rapid, intense enhancement, confirming high vascularity, while fat-suppressed sequences showed no signal loss, excluding macroscopic fat. Phase-sensitive inversion recovery late gadolinium enhancement images from cardiac MRI showed complete gadolinium enhancement of the IVC and RA mass, indicating the absence of fibrosis, necrosis, or myocardial infiltration and supporting tissue characterization consistent with tumor rather than bland thrombus. Positron emission tomography (PET)/CT (Figure 6) demonstrated no abnormal fluorodeoxyglucose uptake, making a metabolically active malignancy less likely. Collectively, these multimodality imaging findings enabled an early and accurate diagnosis of intravenous leiomyomatosis.

## Management

**Figure 7:**
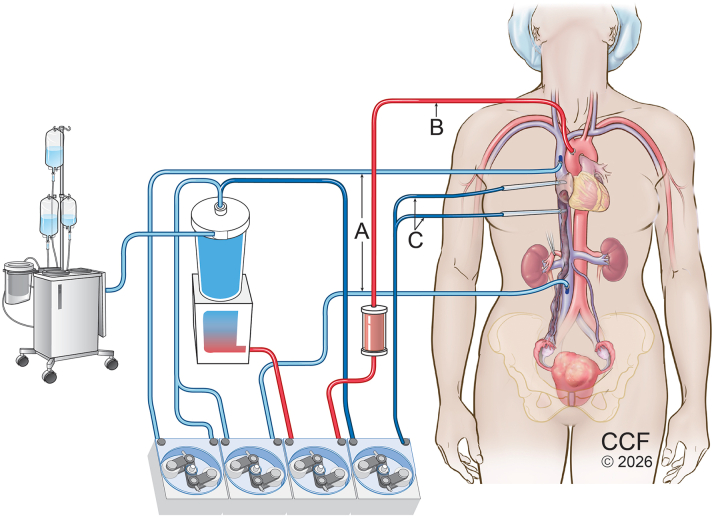
Illustration of Perfusion Technique and Surgical Approach Two venous cannulas (A) and 1 aortic cannula and arterial filter (B). For beating-heart cardiopulmonary bypass, 2 additional pump suckers (C) are used to create a bloodless surgical field. Surgical approach with mid-line incisions of sternum and xiphisternum to pubic bone. Hysterectomy and bilateral salpingo-oopherectomy completed followed by exploratory cardiotomy through right atrium approach and dissection down to IVC. The whole mass was pulled down from the IVC through an incision made above the right gonadal vein. The IVC was repaired with an autologous pericardium patch.

A multidisciplinary team involving cardiothoracic surgery, vascular surgery, urology, and gynecology was consulted to formulate a comprehensive treatment plan. The initial recommendation was to continue anticoagulation for 6 months before definitive surgery, given the initial concern for thrombus. The patient subsequently sought a second opinion at Cleveland Clinic, where a single-stage, en bloc resection was performed (Figure 7). The operation included a total hysterectomy, bilateral salpingo-oophorectomy, laparotomy, and sternotomy under cardiopulmonary bypass, achieving complete removal of the mass. An exploratory cardiotomy was performed through an RA approach, providing direct visualization of the intracardiac component and confirming that the tumor was nonadherent. The tricuspid valve and atrial walls were intact, with no evidence of infection, thrombus, or adhesion. The entire tumor was then extracted through an incision in the anterior surface of the IVC near the insertion of the right gonadal vein. The vena cava was closed with an autologous pericardial patch. The entire mass was excised en bloc without complication.

**Figure 8:**
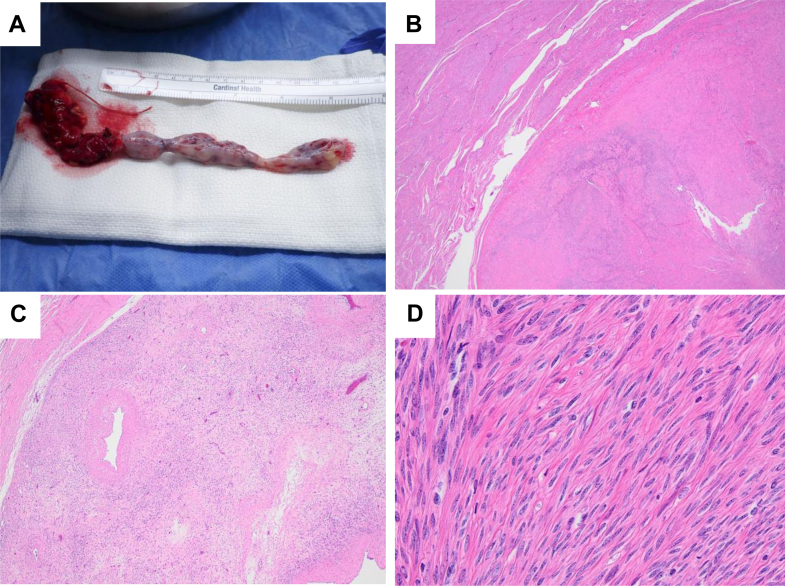
Gross and Histopathologic Features of the Resected Intravascular Leiomyomatosis (A) Gross photograph of the intravascular leiomyomatosis specimen extending from the IVC into the RA, measuring 16.5 cm in total length and 0.6 to 1.7 cm in diameter. The cranial (atrial) extent of the tumor is positioned on the right side of the image. (B to D) Hematoxylin and eosin staining and immunohistochemistry demonstrating smooth-muscle differentiation consistent with leiomyoma. (B) Microscopic image of the IVC mass. (C) Intermediate-power view showing a well-circumscribed lesion composed of bland spindle cells with focal hydropic change and absence of significant atypia or necrosis. (D) Bland spindle-cell proliferation composed of smooth-muscle cells. Immunoperoxidase studies show the neoplastic cells are positive for desmin and SMA, confirming smooth-muscle origin. IVC = inferior vena cava; RA = right atrium; SMA = smooth muscle actin.

Intraoperatively, the tumor was smooth, spongy, and mobile within the IVC and extended from the right gonadal vein into the RA (Figure 8A). Histopathologic examination confirmed a benign leiomyoma, with tumor cells positive for desmin and smooth muscle actin (SMA) (Figures 8B to 8D). Postoperative recovery was uneventful, and follow-up imaging at 6 months showed no recurrence.

## Outcome and Follow-Up

The patient recovered uneventfully and was discharged home in stable condition after a single-stage surgical resection. At the 6- and 12-month follow-up, she remained asymptomatic, and serial CT imaging showed no evidence of recurrence. Long-term surveillance with CT or MRI every 6 to 12 months was recommended. A benign right breast cyst identified during follow-up is being monitored with interval imaging.

## Discussion

IVL/ICL are rare benign smooth-muscle neoplasms originating from the uterine or pelvic veins and characterized by intraluminal extension into the venous system. Although histologically benign, they display biologically aggressive behavior because of continuous intravascular growth and potential extension into the IVC and right heart chambers.^1^

The first case of IVL was described by Birch-Hirschfeld in 1895,^2^ and its cardiac extension was first reported by Dürck in 1907.^3^ Despite growing recognition, IVL/ICL remains rare; a 2022 systematic review identified only 748 reported cases in the English-language literature, most affecting women in their fifth decade of life.^4^ Recently, Wen et al^1^ analyzed 216 patients over 20 years and proposed a refined stage-and-type classification to guide surgical management. Tumor extent is categorized as stage 1 (confined to pelvic veins), stage 2 (extends to the IVC but below the RA), stage 3 (extends into the RA or ventricle but below the pulmonary valve), and stage 4 (involves the pulmonary artery). For stage ≥2 disease, 3 morphologic types are defined: type I (free-floating and nonadherent), type II (adherent to the vessel wall or endocardium), and type III (pedunculated and isolated by a stalk).

Our patient had stage 3 type I disease—tumor extension from the right gonadal vein through the IVC into the RA without wall adhesion—a presentation associated with risk of right-sided heart failure, thromboembolism, and cardiogenic shock.^5^ Early diagnosis is often difficult because clinical manifestations are nonspecific, and intravascular masses are frequently misinterpreted as thrombus or malignant tumor thrombus.

The differential diagnosis for an IVC-RA intraluminal mass includes bland thrombus, malignant tumor thrombus, RA myxoma, and fat-containing masses, each of which carries distinct imaging characteristics and therapeutic implications. Bland thrombus typically shows low vascularity, absent perfusion, and a lack of enhancement—features inconsistent with our patient's highly vascular mass, making prolonged anticoagulation an unlikely effective therapeutic strategy. Malignant tumor thrombus (eg, renal cell carcinoma or uterine leiomyosarcoma) often demonstrates heterogeneous enhancement, wall invasion, and fluorodeoxyglucose uptake, none of which were present. Myxomas usually arise from the interatrial septum rather than track continuously from the pelvic veins, and fat-containing lesions are easily excluded on fat-suppressed sequences. In contrast, IVL is characterized by smooth, continuous venous extension, mobility, high vascularity, and desmin/SMA positivity on pathology—all consistent with the findings in our case.

In this case, the initial CT angiogram suggested thrombosis, leading to anticoagulation and delayed surgical evaluation. Subsequent multimodality imaging—including TTE, CT venography, abdominal and cardiac MRI, PET/CT, and IVUS—was critical for clarification. TTE revealed a mobile intracardiac mass, while CT and MRI demonstrated continuity from the gonadal vein to the RA. IVUS identified multiple intraluminal echolucent channels without endothelial invasion, consistent with a highly vascular intraluminal tumor. Cardiac MRI showed marked vascularization on first-pass perfusion, effectively ruling out thrombus, and PET/CT confirmed the absence of metabolic activity, supporting a benign process. Integrating these complementary findings enabled an early and accurate preoperative diagnosis of intravenous leiomyomatosis originating from the gonadal vein.

These imaging modalities also provided detailed anatomic mapping that enhanced surgical preparedness. As Wen et al^1^ recommended, the standard surgical approach involves laparotomy or thoracolaparotomy with en bloc removal of the tumor thrombus, resection of intravascular components, and total hysterectomy with bilateral salpingo-oophorectomy to eliminate hormonal stimulation. Our patient underwent a complete single-stage excision^6^^,^^7^ under cardiopulmonary bypass, with excellent recovery and no recurrence at 1 year.

Although histologically benign, IVL/ICL can recur, particularly after incomplete resection.^1^^,^^7^ Therefore, long-term surveillance with CT or MRI every 6 to 12 months is essential. Adjuvant hormonal therapy, typically a gonadotropin-releasing hormone agonist^8^ or aromatase inhibitor,^9^ may be considered for patients with residual disease or retained ovarian function, although no standardized guidelines exist and management remains individualized.^1^ Anticoagulation may be used initially when thrombus cannot be excluded, but it does not treat IVL itself.

This case illustrates how the application of a modern stage-and-type framework, combined with comprehensive multimodality imaging, can enable early recognition, precise surgical planning, and excellent clinical outcomes in this rare but potentially life-threatening disease.

## Conclusions

This case underscores the critical role of multimodality imaging in the early and accurate diagnosis of IVL/ICL. Combining TTE, CT, MRI, PET/CT, and IVUS enabled precise anatomic delineation, informed surgical planning, and successful single-stage resection. Although histologically benign, IVL/ICL demonstrates invasive growth potential; therefore, complete excision and long-term imaging surveillance are essential to prevent recurrence.

## Funding Support and Author Disclosures

The authors have reported that they have no relationships relevant to the contents of this paper to disclose.

**Take-Home Messages**

• IVL/ICL should be recognized as a rare but important cause of right-sided cardiac masses, especially in women with a history of uterine leiomyomas, as initial symptoms can vary from asymptomatic findings to signs of venous or cardiac obstruction.
• Multimodality imaging plays a critical role in distinguishing IVL/ICL from thrombus or malignancy, accurately defining disease extent and guiding timely and prompt multidisciplinary surgical planning to achieve complete resection and prevent complications.

## Appendix

Video 1 Modified Parasternal Short-Axis View on TTE

Video 2 RV-Focused Apical 4-Chamber View on TTE: RA Mass Protruded Into the RV in Each Beat

Video 3 Zoomed RV-Focused Apical 4-Chamber View on TTE

Video 4 Sweep of Subcostal View on TTE Demonstrating a Mass Extending From the IVC Into the RA

Video 5 Video of Venogram

Video 6 Intravenous Ultrasound
